# Glucose sensing by carotid body glomus cells: potential implications in disease

**DOI:** 10.3389/fphys.2014.00398

**Published:** 2014-10-15

**Authors:** Lin Gao, Patricia Ortega-Sáenz, María García-Fernández, Patricia González-Rodríguez, Candela Caballero-Eraso, José López-Barneo

**Affiliations:** ^1^Instituto de Biomedicina de Sevilla, Hospital Universitario Virgen del Rocío/Consejo Superior de Investigaciones Científicas/Universidad de SevillaSeville, Spain; ^2^Centro de Investigación Biomédica en Red sobre Enfermedades NeurodegenerativasSeville, Spain; ^3^Departamento de Fisiología Médica y Biofísica, Facultad de Medicina, Universidad de SevillaSeville, Spain; ^4^Unidad Médico-Quirúrgica de Enfermedades Respiratorias, Hospital Universitario Virgen del RocíoSeville, Spain

**Keywords:** carotid body, glucose sensing, O_2_ sensing, hypoglycemia, intermittent hypoxia, sleep apnea, chronic hypoxia, diabetes

## Abstract

The carotid body (CB) is a key chemoreceptor organ in which glomus cells sense changes in blood O_2_, CO_2_, and pH levels. CB glomus cells have also been found to detect hypoglycemia in both non-primate mammals and humans. O_2_ and low-glucose responses share a common final pathway involving membrane depolarization, extracellular calcium influx, increase in cytosolic calcium concentration, and neurotransmitter secretion, which stimulates afferent sensory fibers to evoke sympathoadrenal activation. On the other hand, hypoxia and low glucose induce separate signal transduction pathways. Unlike O_2_ sensing, the response of the CB to low glucose is not altered by rotenone, with the low glucose-activated background cationic current unaffected by hypoxia. Responses of the CB to hypoglycemia and hypoxia can be potentiated by each other. The counter-regulatory response to hypoglycemia by the CB is essential for the brain, an organ that is particularly sensitive to low glucose. CB glucose sensing could be altered in diabetic patients, particularly those under insulin treatment, as well as in other medical conditions such as sleep apnea or obstructive pulmonary diseases, where chronic hypoxemia presents with plastic modifications in CB structure and function. The current review will focus on the following main aspects: (1) the CB as a low glucose sensor in both *in vitro* and *in vivo* models; (2) molecular and ionic mechanisms of low glucose sensing by glomus cells, (3) the interplay between low glucose and O_2_ sensing in CB, and (4) the role of CB low glucose sensing in the pathophysiology of cardiorespiratory and metabolic diseases, and how this may serve as a potential therapeutic target.

## Introduction

Hypoglycemia, or a low blood glucose level, is a physiological condition that is detected by the body to trigger compensatory counter-regulatory responses, which are essential for maintaining glucose supply to organs, such as the brain, strictly dependent on this metabolite for survival. Alterations of glucose sensing might play an important pathogenic role in several diseases, especially those related to sympathoexcitation. The carotid body (CB) is a key chemoreceptor organ that may critically participate in glucose homeostasis. The first study linking the CB to glucose metabolism was reported more than 25 years ago (Alvarez-Buylla and de Alvarez-Buylla, 1988), and knowledge of the molecular mechanism underlying CB glucose sensing has advanced recently due in part to improvements in CB preparations that are suitable for *in vitro* recording of physiological parameters (Pardal and Lopez-Barneo, 2002a). The role of the CB in several cardiorespiratory and metabolic disorders has also been studied in the past few years (Paton et al., 2013; Ribeiro et al., 2013; Schultz et al., 2013) with the CB recently proposed as a potential therapeutic target for these diseases (McBryde et al., 2013).

## Carotid body and O_2_ sensing

The CB, the main arterial chemoreceptor, is located at the carotid artery bifurcation. The CB is composed of clusters (glomeruli) of electrically excitable neuron-like glomus (type I) cells surrounded by glia-like sustentacular (type II) cells. Type II cells, or a subpopulation of them, have recently been identified as neural stem cells that contribute to the growth of the organ in conditions of chronic hypoxemia (Pardal et al., 2007; Platero-Luengo et al., 2014). Type I glomus cells have secretory vesicles containing dopamine and other neurotransmitters. CB glomus cells sense changes in the chemical composition of blood, including O_2_ tension (PO_2_), CO_2_ tension, pH, and other stimuli (reviewed by Lopez-Barneo et al., 2008; Kumar and Prabhakar, 2012).

A major physiological function of the CB is to sense changes in blood PO_2_, as this variable is not detected by central chemoreceptors. CB glomus cells behave as O_2_-sensitive presynaptic-like elements. During hypoxia, O_2_-sensitive K^+^ channels are closed in the plasma membrane of glomus cells, which triggers membrane depolarization, Ca^2+^ influx, and neurotransmitter release. This signal is sent to the brainstem respiratory centers by afferent fibers of the carotid-sinus nerve to mediate a compensatory acute hyperventilatory response in order to increase O_2_ tension in the blood (Weir et al., 2005; Lopez-Barneo et al., 2008). Besides the CB glomus cells, O_2_-sensitive ion channels have been described in numerous cell classes, such as chromaffin cells in the adrenal medulla, neuroepithelial bodies of the lung, pulmonary and systemic vascular smooth muscle, and heart myocytes among others (see for review Lopez-Barneo et al., 1999, 2001).

## Carotid body and glucose sensing

### Glucose sensing in different organs

The brain is very sensitive to decreased glucose supply from the blood. Glucose-sensitive neurons have been found in different regions of the brain (Routh, 2002), including the hypothalamus (Biggers et al., 1989; Dunn-Meynell et al., 2002; Levin et al., 2004; Burdakov et al., 2006) and striatum (Calabresi et al., 1997) to mediate reflexes that counter-balance the changes of glucose level. Glucose-sensitive neurons have specific functional and molecular properties. Glut2, a low-affinity glucose transporter is expressed in some glucose-sensing cells (Schuit et al., 2001; Thorens, 2001). Glucokinase, a low-affinity hexokinase characteristic of pancreatic beta cells, seems to play an important role in both glucose-stimulated and inhibited neurons (Dunn-Meynell et al., 2002). In addition to the well-established role of central neurons in glucose control, numerous pieces of evidence indicate that glucose sensors also exist at the periphery and that they have an essential physiological role (Cane et al., 1986). In addition to α-cells of the pancreas, hypoglycemia-sensitive cells have also been suggested to exist in the liver (Hamilton-Wessler et al., 1994), near the portal vein (Hevener et al., 1997), and in the adrenal gland of the newborn (Livermore et al., 2012).

### Carotid body as a sensor of low glucose

The first evidence linking the CB with glucose metabolism was reported by Alvarez-Buylla and de Alvarez-Buylla (1988), Alvarez-Buylla and Roces de Alvarez-Buylla (1994). More recently, *in vivo* studies demonstrated that the counter-regulatory response to insulin-induced hypoglycemia is impaired in CB-resected dogs (Koyama et al., 2000). Moreover, these animals exhibit suppressed exercise-mediated induction of arterial plasma glucagon and norepinephrine and, therefore, cannot maintain blood glucose levels during exercise (Koyama et al., 2001).

**Figure 1:**
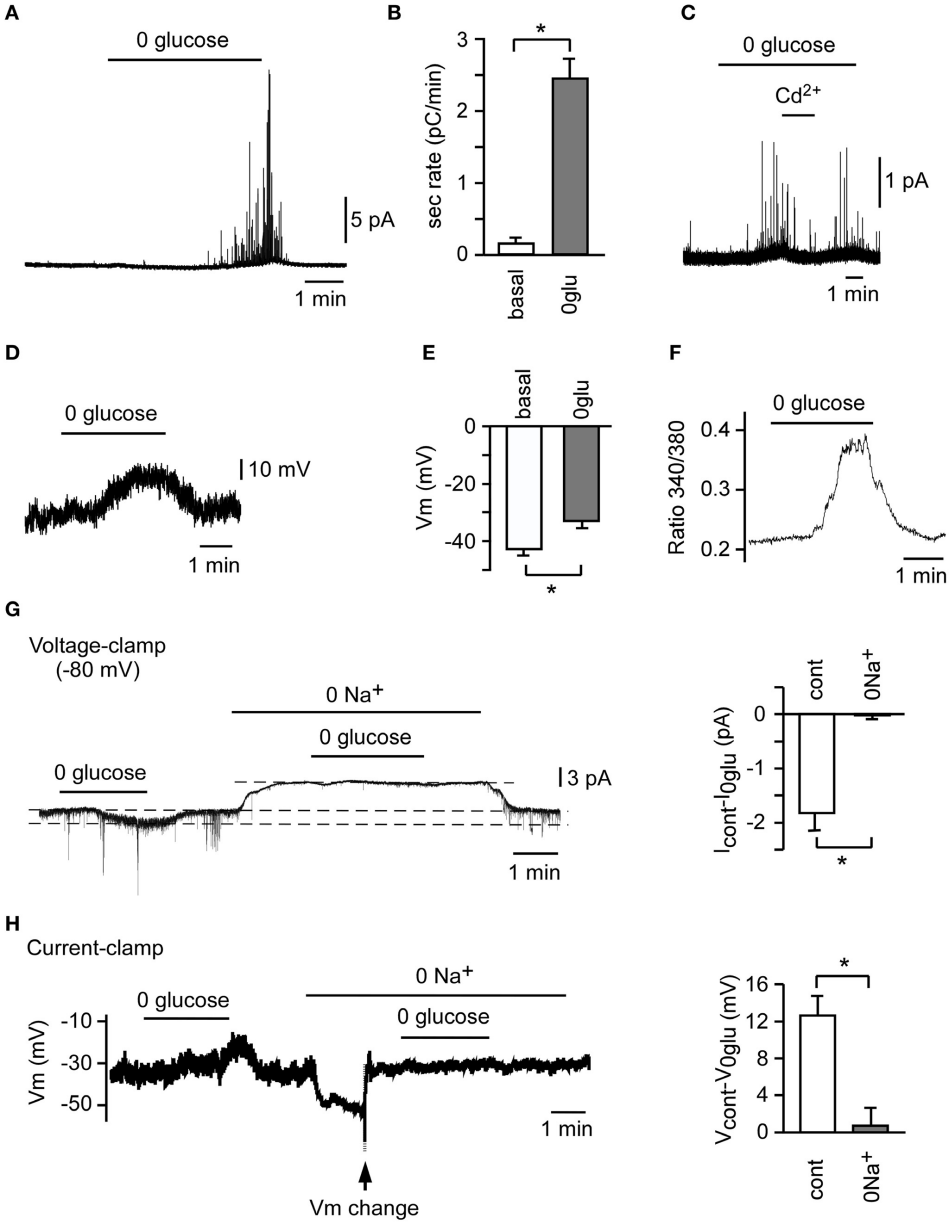
**Counter-regulatory response to hypoglycemia in rat carotid body (CB) slices and isolated glomus cells**. A representative secretory response **(A)** and average secretion rate **(B)** induced by glucopenia in glomus cells from CB slices (*n* = 3). **(C)** Abolition of the secretory response to hypoglycemia by 100 μM Cd^2+^. A representative depolarizing receptor potential **(D)** and average membrane potential **(E)** induced by 0 glucose in CB glomus cells (*n* = 25). **(F)** Reversible increase in cytosolic Ca^2+^ concentration in a Fura-2-loaded glomus cell in response to 0 glucose. **(G)** Abolition of 0 glucose-induced increase in current (Icontrol-I0glu) by replacement of extracellular Na^+^ with N-methyl-D-glucamine (0 Na^+^) in voltage-clamped glomus cells (*n* = 3). **(H)** Inhibition of 0 glucose-induced depolarization (Vcontrol-V0glu) by replacement of extracellular Na^+^ with N-methyl-D-glucamine (0 Na^+^) in current-clamped glomus cells (*n* = 3). To compensate for the hyperpolarization induced by 0 Na^+^, Vm was changed manually to the previous resting value (arrow) ^*^p < 0.05 (Modified from Garcia-Fernandez et al., 2007).

Direct molecular proof of the CB as a glucose-sensing organ was first reported by Pardal and López-Barneo using the CB thin slice preparation and amperometry techniques (Pardal and Lopez-Barneo, 2002b). In this *in vitro* system, rat CB glomus cells secrete neurotransmitter when exposed to a glucose-free solution (Figures 1A,B) (Garcia-Fernandez et al., 2007). This secretory activity is reversible, depending on external Ca^2+^ influx (Figure 1C), and is proportional to the degree of glucopenia. Responses to hypoglycemia, including neurotransmitter release and sensory fiber discharge, have also been observed in other *in vitro* studies using rat CB slices (Garcia-Fernandez et al., 2007; Zhang et al., 2007), rat CB/petrosal ganglion co-culture (Zhang et al., 2007), and cat CB (Fitzgerald et al., 2009). Recently, the hypoglycemia-mediated secretory response has also been detected in human glomus cells dispersed from post mortem CBs (Ortega-Saenz et al., 2013) (see below). However, this topic is controversial as other groups have failed to detect glucose sensing by explanted CBs or dissociated rat CB cells (Bin-Jaliah et al., 2004; Gallego-Martin et al., 2012). Bin-Jaliah et al. (2004) reported CB stimulation in rats secondary to insulin-induced hypoglycemia. However, they proposed that sensing of hypoglycemia by the CB could be an indirect phenomenon dependent on other metabolically mediated blood borne factor. Systemic studies performed in humans have also reported opposing results regarding the role of the CB in hormonal counter-regulatory responses to hypoglycemia (Ward et al., 2009; Wehrwein et al., 2010). Although not fully understood, these discrepancies could possibly result from differences in CB sample preparation or limitations in experimental design. In any event, taken together the available experimental data suggests that low glucose sensing by CBs is likely to be a general phenomenon among mammals that has potential pathophysiological implications.

### Molecular and ionic mechanisms of low glucose sensing by carotid body glomus cells

The molecular mechanisms underlying CB glomus cell activation by hypoglycemia have been investigated in both lower mammals and human CB tissue samples (Pardal and Lopez-Barneo, 2002b; Garcia-Fernandez et al., 2007; Zhang et al., 2007; Fitzgerald et al., 2009; Ortega-Saenz et al., 2013). In our initial study we reported that, like O_2_ sensing by the CB, macroscopic voltage-gated outward K^+^ currents are inhibited in patch-clamped rat glomus cells exposed to glucose-free solutions (Pardal and Lopez-Barneo, 2002b). However, we soon realized that besides this phenomenon, low glucose elicits a membrane depolarization of ~8 mV (Figures 1D,E) (Garcia-Fernandez et al., 2007), which is the main process leading to extracellular Ca^2+^ influx into glomus cells, as demonstrated by microfluorimetry experiments using Fura-2-AM labeled cells (Figure 1F) (Pardal and Lopez-Barneo, 2002b; Garcia-Fernandez et al., 2007; Ortega-Saenz et al., 2013). The increase in intracellular Ca^2+^, which is demonstrated by the inhibition of the secretory activity by Cd^2+^, a blocker of voltage-gated Ca^2+^ channels (Pardal and Lopez-Barneo, 2002b; Garcia-Fernandez et al., 2007), results in exocytotic neurotransmitter release (Pardal and Lopez-Barneo, 2002b; Garcia-Fernandez et al., 2007; Zhang et al., 2007; Ortega-Saenz et al., 2013). This neurotransmitter release triggers afferent discharge and activation of counter-regulatory autonomic pathways to increase the blood glucose level (Zhang et al., 2007; Fitzgerald et al., 2009). The depolarizing receptor potential triggered by low glucose has a reversal potential above 0 mV and is due to the increase of a standing inward cationic current (carried preferentially by Na^+^ ions) present in glomus cells (Figures 1G,H) (Garcia-Fernandez et al., 2007). Indeed, in contrast with hypoxia, low glucose decreases the membrane resistance of glomus cells recorded with the perforated patch configuration of the patch clamp technique to ~50% of control (González-Rodríguez and López-Barneo, unpublished results). As reported by others (Carpenter and Peers, 2001), the background Na^+^ current plays a major role in chemotransduction by glomus cells as it sets the membrane potential to relatively depolarized levels, near the threshold for the opening of Ca^2+^ channels.

### Glucose transport and metabolism in the carotid body during low glucose sensing

The mechanism of low glucose sensing by CB glomus cells does not seem to be the same as high glucose sensing by other glucose-sensing cells in terms of glucose transport and metabolism. Glut2 and glucokinase, molecules specifically expressed in high glucose-sensing cells (Schuit et al., 2001; Thorens, 2001), are not expressed in the CB (Garcia-Fernandez et al., 2007). However, glucose metabolism appears to be necessary for low glucose sensing by the CB, since non-metabolizable glucose fails to prevent the glucose deficiency-induced catecholamine secretion by glomus cells (Garcia-Fernandez et al., 2007).

## Regulation of carotid body low glucose sensing

### Similarities and differences between low glucose and O_2_ sensing

**Figure 2:**
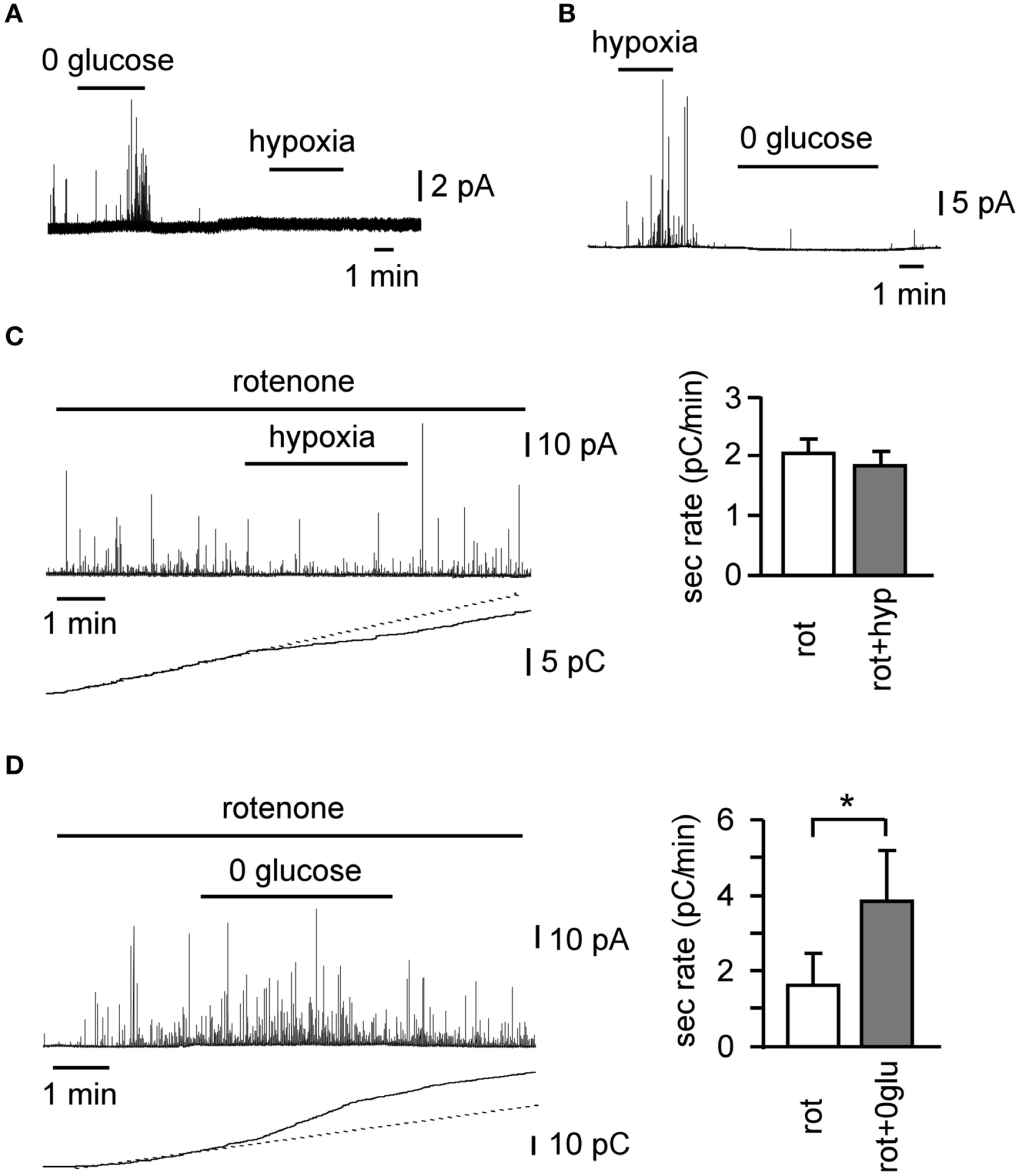
**Differential sensitivity of glomus cells to oxygen and low glucose in rat carotid body slices. (A,B)** Examples of cells with differential secretory responses to hypoxia and low glucose. Differential effect of 100 nM rotenone on the secretory response induced by hypoxia **(C)** (*n* = 14) and hypoglycemia **(D)** (*n* = 5), as demonstrated by a representative amperometric recording, cumulative secretion signal, and average secretion rate. ^*^p < 0.05 (Modified from Garcia-Fernandez et al., 2007).

O_2_ and low-glucose sensing by the CB share many similarities. Both signaling pathways involve the inhibition of voltage-gated K^+^ channels, plasma membrane depolarization, influx of extracellular Ca^2+^, neurotransmitter release, and afferent nerve firing to transmit the signal to the brain, in order to trigger counter-regulatory responses to increase blood O_2_ tension and glucose concentration. On the other hand, the initial steps of the signaling pathways are different for each. Low glucose triggers a depolarizing receptor potential, which is dependent on the activation of background cationic Na^+^-permeable channels (Garcia-Fernandez et al., 2007), which do not seem to be regulated by hypoxia (Carpenter and Peers, 2001). Although voltage-gated K^+^ channels are inhibited upon exposure of CB glomus cells to low glucose, this inhibition has a minimal effect regarding neurotransmitter secretion (Garcia-Fernandez et al., 2007). Indeed, as stated above, low glucose induces a decrease in the input resistance of cells, whereas the predominant effect of hypoxia is an increase in input resistance. Although glomus cells normally secrete neurotransmitters in response to glucose and hypoxia, there are cells that respond to only one of these two stimuli (Figures 2A,B). Moreover, rotenone, a specific mitochondrial complex I inhibitor, which blocks hypoxia-induced catecholamine secretion (Ortega-Saenz et al., 2003), shows no effect on the low glucose-induced secretory activity in CB cells (Figures 2C,D) (Garcia-Fernandez et al., 2007). Therefore, it appears that sensitivities to hypoglycemia and hypoxia depend on separate signal transduction mechanisms, although they share the same final steps leading to transmembrane Ca^2+^ influx and neurotransmitter release. The mechanism of CB O_2_ sensing is as yet unknown; however a considerable body of knowledge including our rotenone data, suggests that mitochondria may play an important direct or indirect role (Ortega-Saenz et al., 2003; see Buckler and Turner, 2013 for an update and references). The fact that rotenone does not alter glomus cell responses to hypoglycemia indicates that low glucose sensing is not related to oxidative phosphorylation and could depend on metabolites of the glycolytic pathway (Garcia-Fernandez et al., 2007).

### Interplay between low glucose and O_2_ sensing

**Figure 3:**
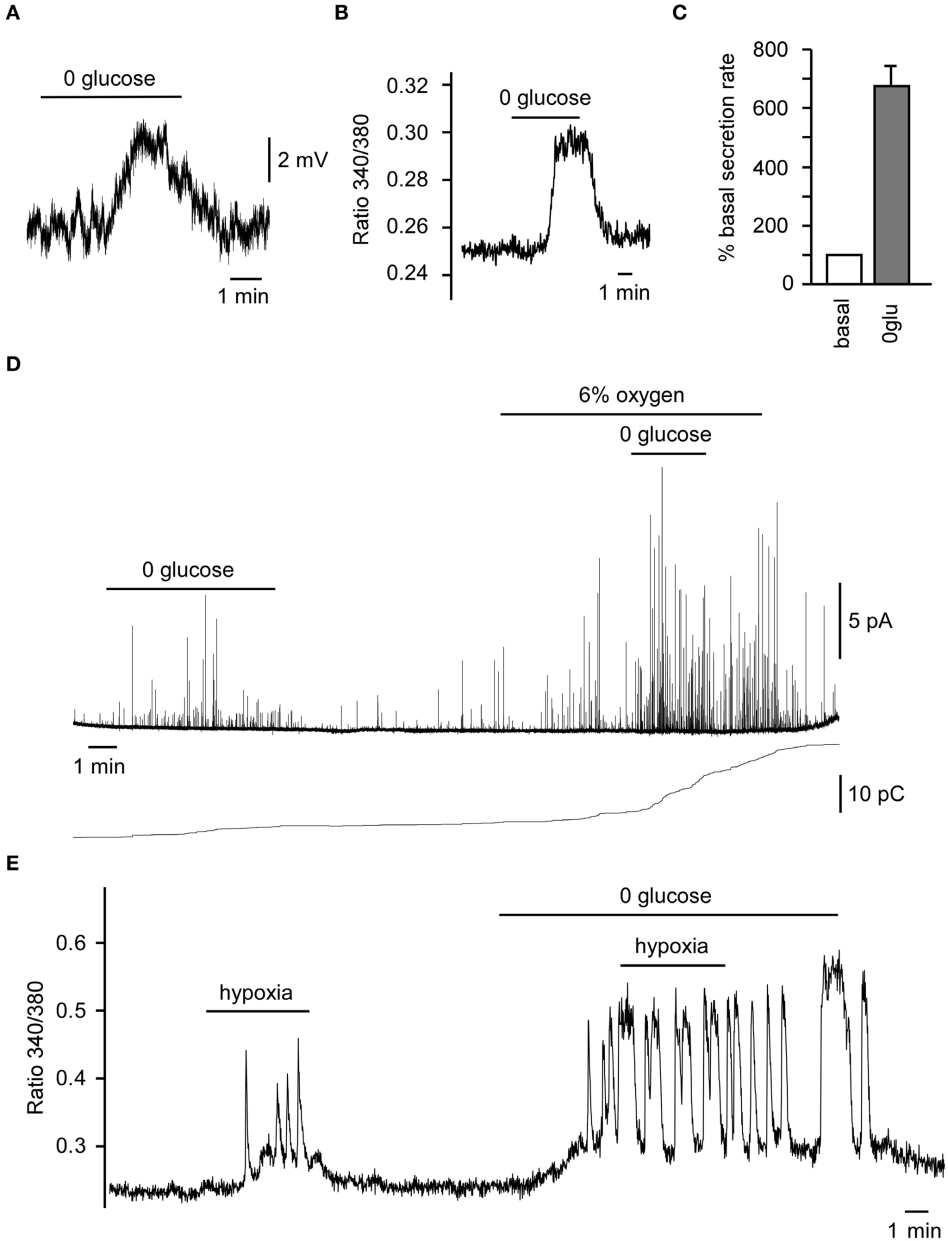
**Responses of human carotid body (CB) glomus cells to low glucose and hypoxia**. (**A**) Depolarizing receptor potential recorded in a current-clamped human glomus cell in response to glucopenia. **(B)** Reversible increase in cytosolic Ca^2+^ in a Fura-2-loaded glomus cell exposed to 0 glucose. **(C)** Average secretion rate induced by hypoglycemia (*n* = 2). **(D)** Secretory response to 0 glucose of glomus cells in CB slices and the potentiation of the 0 glucose-induced secretory response by mild hypoxia (6% O_2_) as demonstrated by a representative amperometric recording (top) and cumulative secretion signal (bottom). **(E)** Representative recording of a reversible increase of cytosolic Ca^2+^ in a Fura-2-loaded glomus cell, demonstrating the potentiation of the hypoxic-response by hypoglycemia. Modified from Ortega-Saenz et al. (2013).

The brain is very sensitive to decreases both in arterial O_2_ tension and glucose level. Being a polymodal sensor of O_2_, glucose, pH, CO_2_, etc., a coordinated response to hypoxia and hypoglycemia by CB chemoreceptors could prevent to a major extent the detrimental effects caused by both conditions. Although a small percentage of CB glomus cells respond specifically to only hypoxia or low glucose (Garcia-Fernandez et al., 2007), in a majority of glomus cells hypoxia and hypoglycemia can potentiate each other's response, such as is seen with neurotransmitter release and afferent discharge (Pardal and Lopez-Barneo, 2002b; Zhang et al., 2007; Fitzgerald et al., 2009). The secretory response to low glucose increases in the presence of low PO_2_ in rat CB slices (Pardal and Lopez-Barneo, 2002b), and we have recently shown that glomus cells in the human CB are also glucose sensors and show the same responses (cell depolarization, increased cytosolic Ca^2+^ and neurotransmitter secretion), as described in lower mammals (Figures 3A–D). In this preparation, hypoxia (6%O_2_) potentiates low glucose-induced catecholamine secretion, whereas low glucose further induces Ca^2+^ influx during hypoxia (Figures 3D,E). The effect of hyperoxia on hypoglycemia and the effect of hyperglycemia on hypoxia are less well known. A recent human study suggested that hyperoxia could blunt the hypoglycemia effect (Wehrwein et al., 2010). Another study suggested that both hypo and hyperglycemia could increase the hypoxic response in human subjects (Ward et al., 2007).

### Intermittent hypoxia and glucose sensing

No direct evidence has been reported regarding the effect of intermittent hypoxia on glucose sensing by the CB. In rat CB glomus cells, intermittent hypoxia enhances acute hypoxia-induced membrane depolarization and the inhibition of TASK-like K^+^ channels (Ortiz et al., 2013). Intermittent hypoxia has also been found to augment the CB sensory response to acute hypoxia and to enhance the hypoxic ventilatory chemoreflex in neonatal rats (Peng et al., 2004). However, a recent study reported an exaggerated activation of CB afferent activity accompanied by hypoventilation in a rat model of intermittent hypoxia when exposed to acute hypoxia (Gonzalez-Martin et al., 2011). It is logical to speculate that intermittent hypoxia could potentiate the carotid chemoreceptor response to hypoglycemia, as occurs with hypoxia. Indeed, intermittent hypoxia has been found to be associated with altered glucose metabolism in rodent models. Intermittent hypoxia results in an increase in fasting glucose and a decrease in insulin level in neonatal rats, which is associated with a disturbed glucose homeostasis (Pae et al., 2013). In mouse, intermittent hypoxia triggers increased fasting glucose and decreased sensitivity to insulin, with the former being reversed by discontinuation of exposure to hypoxia (Polak et al., 2013). Few human studies have been carried out to study the relationship between intermittent hypoxia and glucose homeostasis. Individuals exposed to intermittent hypoxia demonstrate an increased sympathetic nerve activity (Cutler et al., 2004), while male adults exposed to high altitude hypoxia have decreased insulin sensitivity (Larsen et al., 1997).

### Insulin and carotid body glucose sensing

In addition to hypoxia and intermittent hypoxia, insulin was found recently to be a regulator of the CB response to hypoglycemia. Indeed, insulin was proposed as a new intermittent hypoxia-like agent, and carotid chemoreceptors have been suggested to contribute to insulin-mediated sympathoexcitation (Limberg et al., 2014). Animal studies indicate that CB cells have insulin receptors and respond to increases in insulin levels by inducing sympathetic activation, as demonstrated by altered arterial blood pressure, breathing, and neurotransmitter release (Bin-Jaliah et al., 2004; Ribeiro et al., 2013). The combined activation of CB chemoreceptors by insulin and low glucose may serve as a counter-balance mechanism to limit the decrease of glucose levels in insulin-treated patients. In this regard, it would be interesting to explore whether long-lasting CB exposure to high glucose, as occurs in diabetic patients, alters the low glucose sensitivity of glomus cells.

## Carotid body dysfunction in disease states

CB acts as a combined oxygen and glucose sensor to facilitate activation of the counter-regulatory measures in response to small reductions of either variable. Such measures include, on one hand, hyperventilation and increased blood pressure to facilitate blood-borne O_2_ supply to organs and, on the other hand liver glycogenolysis and insulin resistance of peripheral tissues to combat hypoglycemia. Diseases altering the structure and function of CB chemoreceptors could have detrimental effects, leading to dysregulation of glucose homeostasis.

### Obstructive sleep apnea

Obstructive sleep apnea (OSA) is a common clinical syndrome characterized by intermittent hypoxia and sleep fragmentation. OSA is a well-established significant risk factor for cardiovascular disease and mortality. As indicated above Intermittent Hypoxia and Glucose Sensing, chronic intermittent hypoxia results in CB chemoreceptor over-stimulation and augmentation of CB sensory responses in rats (Peng et al., 2003) and humans (Cutler et al., 2004). Intermittent hypoxia has been found to be associated with altered glucose metabolism and insulin resistance in rodent models (Pae et al., 2013; Polak et al., 2013), but its effects on glucose homeostasis in humans are as yet unstudied. It can be expected that CB overstimulation and growth seen in OSA patients (Nair et al., 2013; Abboud and Kumar, 2014) should lead to hyperglycemia and over-sensitivity to low glucose. Nevertheless, O_2_ and glucose act on separate sensing mechanisms in glomus cells and, in addition, OSA can be accompanied by hypertension and diabetes. Therefore, the impact of OSA syndrome on CB-mediated glucose homeostasis requires future studies using human CB tissue samples (Ortega-Saenz et al., 2013).

### Diabetes

Type 2 diabetes is a major chronic disease associated with high morbidity, mortality, and economic burden. Glucose sensing is essential for insulin-treated diabetic patients to counter-regulate insulin-induced hypoglycemia. It has been proposed that the CB dysfunction, increasing sympathetic tone and catecholamines in the blood, could possibly contribute to the pathogenesis of type 2 diabetes and essential hypertension (Nimbkar and Lateef, 2005). Using a computed tomographic angiography technique, enlargement of the CB is observed in patients with diabetes mellitus, hypertension, and congestive heart failure relative to controls, which supports the proposed functional relationship between the CB and sympathetically mediated disease states (Cramer et al., 2014). In insulin-dependent diabetic rats, the CB volume is increased, due to an increase in the extravascular volume (Clarke et al., 1999). It is still unclear whether the CB enlargement is a cause of diseases or a consequence of disease progression. Whether CB glucose sensing is altered in diabetic patients is also unknown (see below).

### Relationship between obstructive sleep apnea and diabetes

OSA syndrome and type 2 diabetes are also strongly linked to each other. Patients with OSA have an increased incidence of impaired glucose metabolism and are at an increased risk of developing type 2 diabetes (Tasali et al., 2008). On the other hand, the majority of patients with type 2 diabetes also have OSA (Tasali et al., 2008). Although the mechanism is most likely multifactorial, chronic intermittent hypoxia experienced by OSA patients could trigger CB chemoreceptor over-activity, leading to insulin resistance and abnormal glucose metabolism (Tasali et al., 2008). Indeed, insulin resistance is developed in both lean mice (Iiyori et al., 2007) and genetically obese mice (Polotsky et al., 2003) treated with intermittent hypoxia. The secretory activity of the CB is increased in the insulin-resistant rat model, whereas carotid sinus nerve resection prevents CB over-activation and diet-induced insulin resistance (Ribeiro et al., 2013). Therefore, sympathoexcitation due to CB over-stimulation could play an important role in the pathogenesis of both OSA and type 2 diabetes.

## Conclusions

Carotid chemoreceptors work in coordination with other glucose sensing organs to counter-regulate hypoxia and hypoglycemia. The responses to hypoxia and hypoglycemia could be potentiated by each other. Failure to respond to these stresses could lead to malfunction of organs, such as the brain, which is highly sensitive to glucose and O_2_ levels. Indeed, defects in CB function have been associated with several respiratory disturbances, particularly in the newborn (reviewed by Lopez-Barneo et al., 2008). CB over-stimulation could also exert detrimental effects, as has been demonstrated in OSA, hypertension and type 2 diabetes. However, whether the intrinsic glucose responsiveness of glomus cells is altered in these diseases is yet to be determined. Due to the essential role of the CB in sympathetic activation, this organ could serve as a potential therapeutic target for diseases with sustained hyperinsulinemia and sympathoexcitation, such as obesity, hypertension, sleep apnea, metabolic syndrome, cardiovascular disease, and diabetes (Paton et al., 2013). Evaluation of CB size in these conditions can be now studied with noninvasive computed tomography angiography (Nair et al., 2013; Cramer et al., 2014). However, bilateral surgical ablation of the CB performed in asthmatic patients or during neck tumor surgery causes permanent abolition of the ventilatory response to hypoxia. In addition, this condition causes a decrease in the CO_2_ sensitivity of the respiratory center and, in some cases, long term resting hypoventilation and hypercapnia (reviewed by Timmers et al., 2003, see also Dahan et al., 2007). The counter-regulatory response to hypoglycemia could be also altered in patients who have had their CB removed, a status particularly critical in diabetic patients subjected to insulin treatment and therefore at high risk of hypoglycemia. Unilateral CB resection appears to be well tolerated (reviewed by Timmers et al., 2003, see also Minguez-Castellanos et al., 2007), thus making this likely to be a safer therapeutic option. Ideally, new reversible pharmacological tools should be developed to inhibit CB function. In this regard, selective inhibition of the O_2_-sensing mechanisms or CB growth in chronic hypoxia (Platero-Luengo et al., 2014) could reduce CB over-activation while maintaining intact the counter-regulatory response to low glucose.

### Conflict of interest statement

The authors declare that the research was conducted in the absence of any commercial or financial relationships that could be construed as a potential conflict of interest.
